# Efficacy of photodynamic therapy combined with minocycline for treatment of moderate to severe facial acne vulgaris and influence on quality of life

**DOI:** 10.1097/MD.0000000000009366

**Published:** 2017-12-22

**Authors:** Xinghua Xu, Yi Zheng, Zigang Zhao, Xin Zhang, Pengxiang Liu, Chengxin Li

**Affiliations:** a Department of Dermatology; b Department of Neurosurgery, Chinese PLA General Hospital; c Department of Dermatology, Beijing Chao-yang Hospital, Capital Medical University, Beijing, China.

**Keywords:** acne vulgaris, minocycline, photodynamic therapy, quality of life

## Abstract

**Background:**

Acne vulgaris is a prevalent skin disorder impairing both physical and psychosocial health. This study was designed to investigate the effectiveness of photodynamic therapy (PDT) combined with minocycline in moderate to severe facial acne and influence on quality of life (QOL).

**Methods:**

Ninety-five patients with moderate to severe facial acne (Investigator Global Assessment [IGA] score 3–4) were randomly treated with PDT and minocycline (n = 48) or minocycline alone (n = 47). All patients took minocycline hydrochloride 100 mg/d for 4 weeks, whereas patients in the minocycline plus PDT group also received 4 times PDT treatment 1 week apart. IGA score, lesion counts, Dermatology Life Quality Index (DLQI), and safety evaluation were performed before treatment and at 2, 4, 6, and 8 weeks after enrolment.

**Results:**

There were no statistically significant differences in characteristics between 2 treatment groups at baseline. Minocycline plus PDT treatment led to a greater mean percentage reduction from baseline in lesion counts versus minocycline alone at 8 weeks for both inflammatory (−74.4% vs −53.3%; *P* < .001) and noninflammatory lesions (−61.7% vs −42.4%; *P* < .001). More patients treated with minocycline plus PDT achieved IGA score <2 at study end (week 8: 30/48 vs 20/47; *P* < .05). Patients treated with minocycline plus PDT got significant lower DLQI at 8 weeks (4.4 vs 6.3; *P* < .001). Adverse events were mild and manageable.

**Conclusions:**

Compared with minocycline alone, the combination of PDT with minocycline significantly improved clinical efficacy and QOL in moderate to severe facial acne patients.

## 1 Introduction

Acne vulgaris is a chronic inflammatory disease of the pilosebaceous unit resulting from androgen-induced increased sebum production, altered keratinization, inflammation, and bacterial colonization of hair follicles on the face, neck, chest, and back by Propionibacterium acnes. The clinical features of acne include seborrhoea (excess grease), noninflammatory lesions (open and closed comedones), inflammatory lesions (papules and pustules), and various degrees of scarring. It affects 85% of young people (aged 12–24 years), and may persist into their thirties in up to 40% of those affected.^[1,2]^ Acne results in physical symptoms such as soreness, itching, and pain, but its main effects are on quality of life (QOL). Studies assessing the effect of acne, especially facial acne, on psychological health, found a range of abnormalities including depression, lack of self-confidence, suicidal ideation, anxiety, psychosomatic symptoms, low self-esteem, embarrassment, and social inhibition, which improve with effective treatment.^[3–5]^

Several forms of treatment have been offered for moderate to severe acne including topical and systemic medications. Oral isotretinoin is the current mainstay of systemic therapy; however, it has acknowledged adverse reactions, teratogenicity, and psychological effects, which may restrict its use.^[6]^ Though controversial, oral antibiotics are still first-line choice in patients with severe inflammatory acne.^[7]^ Minocycline is a semisynthetic, second-generation tetracycline used for a variety of infectious diseases and acne. It is considered to have a superior efficacy in the treatment of inflammatory acne.^[8]^

Photodynamic therapy (PDT) is an emerging modality in the treatment of acne, in which photosensitizers are applied to the skin to produce reactive oxygen species, and, in the setting of light activation, destroy cells that have absorbed the photosensitizer.^[9]^ Aminolevulinic acid (ALA), methyl aminolevulinate (MAL), and, more recently, indole-3-acetic acid (IAA) have been studied as photosensitizers for PDT.^[10]^ Developed in recent years, ALA is not a photosensitizer, but rather a metabolic precursor substance of protoporphyrin IX, which is a photosensitizer.^[11]^ ALA with red light has been shown to have some selectivity for sebaceous glands and can reduce inflammatory lesion counts by suppressing sebum production.^[12]^ Nevertheless, PDT is also commonly associated with adverse events such as pain, erythema, swelling, and dyspigmentation, which may impair patients’ QOL. In this study, we investigated the efficacy of PDT combined with minocycline and the influence on QOL in patients with moderate to severe facial acne in comparison with that of minocycline alone.

## 2 Methods

### 2.1 Patients

This was a prospective study conducted at Department of Dermatology, Chinese PLA General Hospital, between July 2015 and November 2016. Male and female patients aged 15 to 35 years with moderate to severe facial acne vulgaris were enrolled. Moderate to severe acne was defined by the Investigator Global Assessment (IGA) scale of 3 or 4.^[13]^ Patients were to have ≥10 inflammatory lesions (papules, pustules, or nodules) and ≥10 noninflammatory lesions (open and closed comedones) on the face. The exclusion criteria were: acne fulminans, acne conglobata, secondary acne, or dysplastic naevi in the treatment area; systemic acne treatment with oral isotretinoin within 6 months or oral antibiotics in the past 1 month; history of facial procedures like dermabrasion, chemical, or laser peels; phototherapy within 1 month; topical treatments other than medicated cleansers within 14 days; female patients who were pregnant or nursing; history of photosensitive diseases, porphyria, or porphyrin sensitivity.

**Figure 1:**
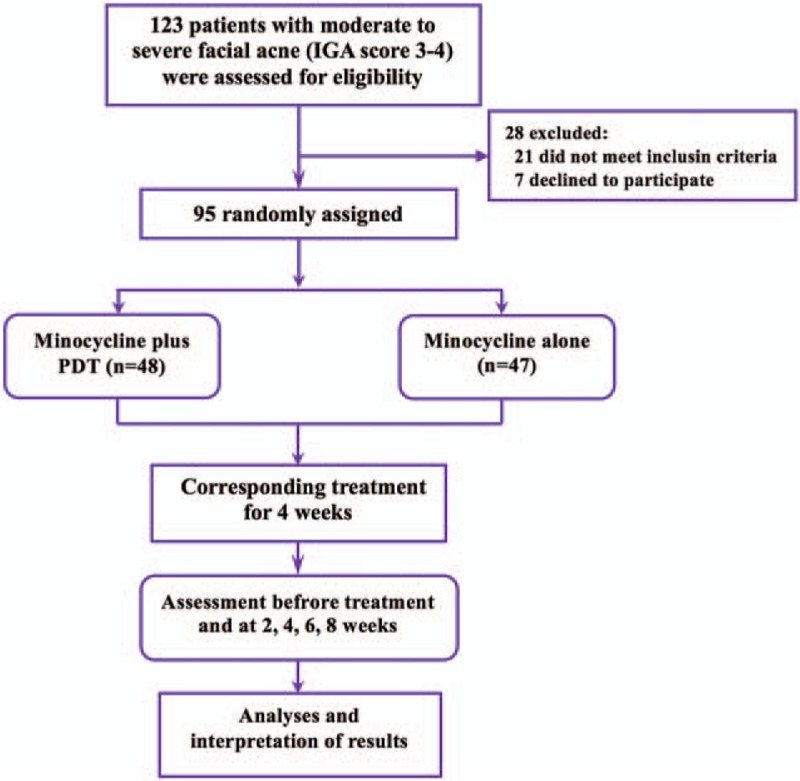
Flowchart of the study. In all, 95 patients were recruited in the study.

The study flowchart is shown in Fig. 1. A total of 95 patients were randomly assigned to the minocycline plus PDT group or minocycline-alone group using computer-generated random numbers, which were packaged in envelopes. This study was approved by the Institutional Review Board of Chines PLA General Hospital and was conducted in conformity to the Declaration of Helsinki. All patients gave written informed consents before enrolling in this study; for juveniles, their parent(s) gave the consent.

### 2.2 Treatment procedures

Eligible patients in the minocycline group took the minocycline hydrochloride capsule (Wyeth Pharmaceutical Co. Ltd., Philadelphia, PA) 100 mg/d for 4 weeks,^[14]^ whereas patients in the minocycline plus PDT group received PDT apart from taking minocycline as patients in the minocycline group. Patients received 4 PDT treatments once a week at weeks 0, 1, 2, and 3. Evaluations were conducted before study, and 2, 4, 6, and 8 weeks after first treatment. 5% ALA solution (50 mg/g) was freshly prepared with ALA powder (Fudan-Zhangjiang Bio-Pharmaceutical Co., Ltd., Shanghai, China). Before ALA application, the skin was cleaned with 70% isopropyl alcohol. Then, fresh 5% ALA solution was applied evenly to the face (from above the jawline, but keeping off the periocular area, including the upper eyelid) and the application site was covered with an occlusive, nonabsorbent dressing. Patients were advised to avoid exposure to sunlight or bright indoor light during incubation. The duration of incubation was 90 minutes.

After incubation, the cream was gently cleaned off with saline and the area was illuminated with 633-nm red light originated from a light-emitted diode light source (Wuhan Yage Optic and Electronic Technique Co. Ltd., Wuhan, China) for 20 minutes at an output power density 100 mW/cm^2^, with a final light dose of 120 J/cm^2^.^[15]^ Output power density was set at 100 mW/cm^2^ at the very beginning, and when the patient could not tolerate because of pain, short pauses were included. If the patient still could not tolerate, the therapist would turn the power density down to 40 mW/cm^2^ and increased the power density by 20 mW/cm^2^ every 5 minutes. Patients and therapists wore specialized protective eyeglasses during illumination. Clinical photographs were taken before treatment and at follow-ups.

### 2.3 Outcome measures

Efficacy was assessed by the IGA score, standardized counts of inflammatory and noninflammatory lesions, and reduction rate of acne lesions, performed by the same investigator for all patients. Reduction rate was calculated as follows: reduction rate (%) = (number of lesions before treatment − number of lesions after treatment)/number of lesions before treatment × 100%. We defined treatment success as an improvement of at least 2 grades (mild, almost clear, or clear) of the IGA score from baseline. Photographs taken before each treatment, 2 days after the first treatment and at follow-up visits, were supportive of efficacy. Adverse reactions were recorded during each treatment, before the next treatment, and in the subsequent follow-up period. The adverse reactions include itching, pain, dizziness, headache, pustules, blisters, edematous erythema, pigmentation, reactive acne, and desquamation.

The Dermatology Life Quality Index (DLQI) was used to investigate how patients lived with facial acne before treatment and the changes in their QOL after treatment at 2, 4, 6, and 8 weeks of treatment beginning.^[16,17]^ The DLQI was a questionnaire comprised of 10 questions to measure the impact of skin disease on the QOL of an affected person. The DLQI score was calculated by summing the score of each question, resulting in a possible maximum score of 30 and a minimum of 0. The higher the score, the more the QOL is impaired.

Observed and spontaneously reported adverse events (before and after illumination, and at follow-ups) were recorded, with onset, severity, and duration. As pain was expected adverse events to PDT, patients assessed pain intensity felt immediately after illumination using the visual analog scale where 0 represented no pain and 10 represented worst severe pain. If severe erythema occurred, the patient would be advised to protect the face from sunlight or intense light, and compress the face with cold saline. The PDT treatment interval would be extended accordingly.

### 2.4 Statistical analysis

The study was analyzed on an intention-to-treat basis, and all statistical analyses were performed with SPSS statistics 22.0 (IBM Corporation). Statistical diagrams were drawn by GraphPad Prism 6 (GraphPad Software). After conformation of normal distribution, data were expressed as mean ± SD and an unpaired *t* test or chi-square test was used for appropriate comparison between 2 groups. All statistical tests were bilateral, with a significance level of 0.05.

## 3 Results

In all, 95 consecutive patients were recruited in the study: 48 in the minocycline plus PDT group and 47 in the minocycline group. Patients’ characteristics at baseline are summarized in Table 1. The study population (median age 24 years, range 15–35) presented slightly more females (56.8%) than males. The mean course of disease was 11.0 ± 3.8 months. At baseline, there were no statistically significant differences between 2 treatment groups in terms of age, sex, IGA score, acne lesion counts, and course of disease.

**Table 1:**
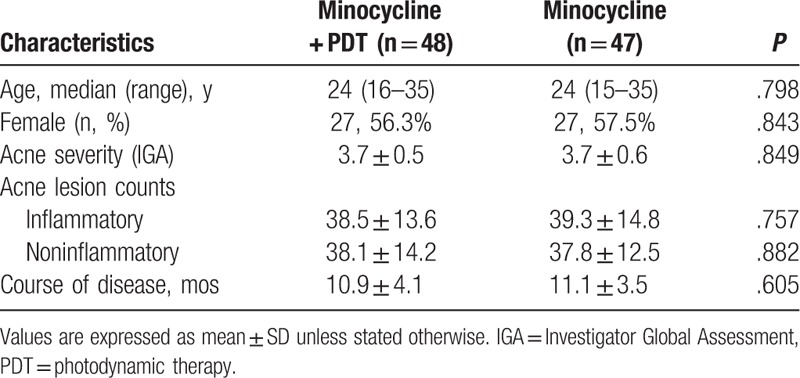
Patients’ demographics and clinical characteristics at baseline.

### 3.1 Efficacy

**Figure 2:**
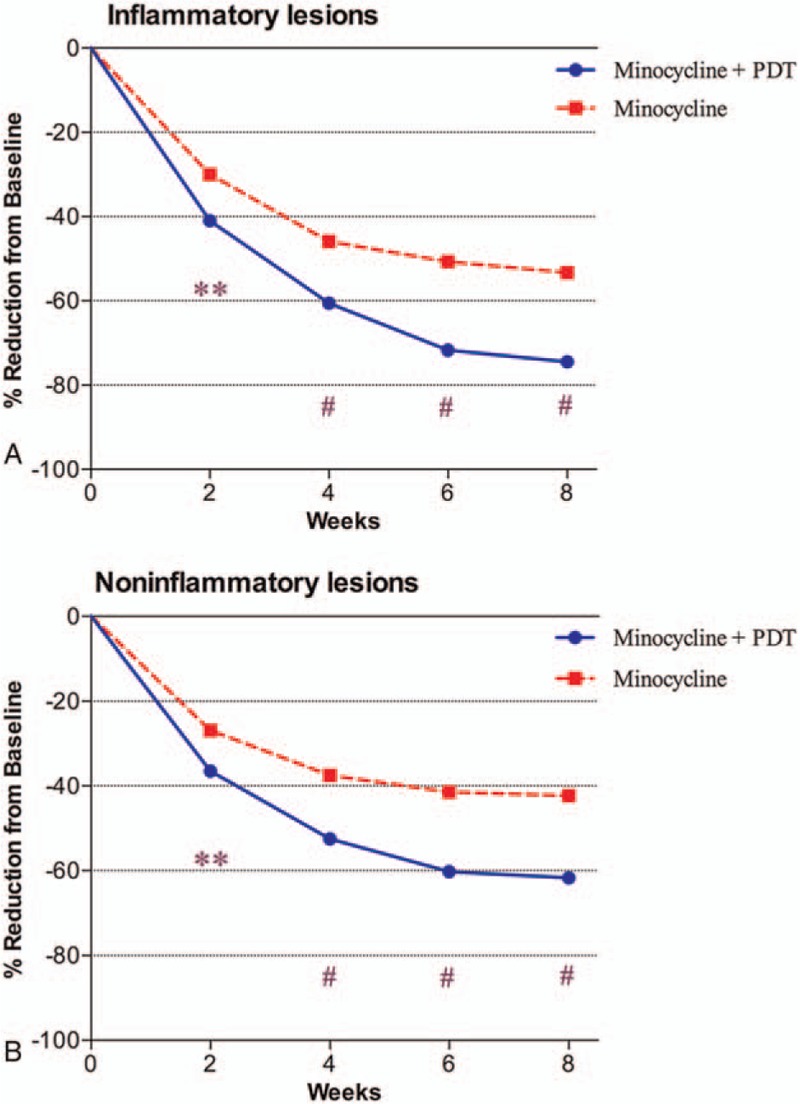
Mean percentage changes in (A) inflammatory lesion counts and (B) noninflammatory lesion counts from baseline to each study visit. The red line indicates minocycline plus PDT and the blue line indicates minocycline alone. ^∗∗^*P* < .01, ^#^*P* < .001. PDT = photodynamic therapy.

**Figure 3:**
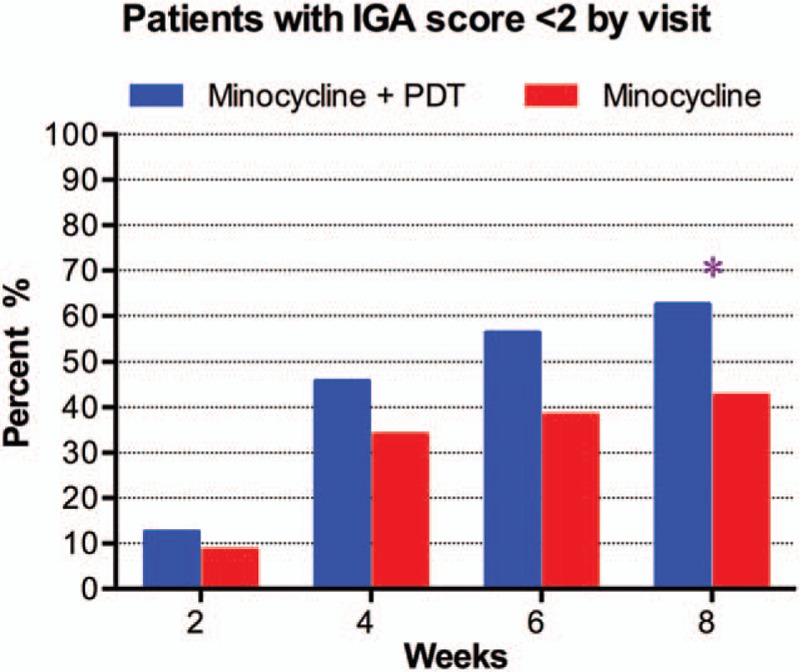
Percentage of patients with Investigator Global Assessment (IGA) of “clear” or “almost clear” (IGA score <2) at each study visit. The proportions are compared using chi-square test. ^∗^*P* < .05.

**Figure 4:**
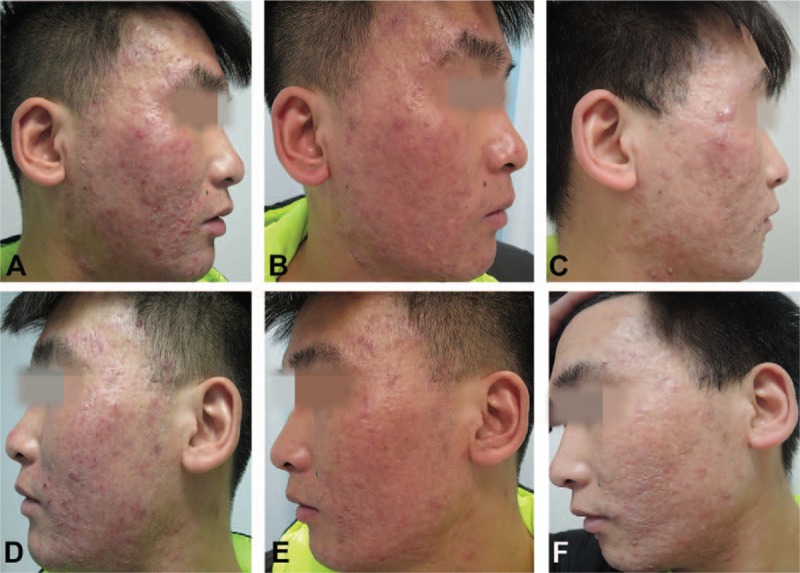
Clinical photographs taken from a patient treated with minocycline plus PDT. (A, D) Before treatment; (B, E) at 2 weeks’ follow-up; (C, F) follow-up after 8 weeks. PDT = photodynamic therapy.

The response to treatment during the study is shown in Fig. 2. Treatment with minocycline and PDT led to a greater mean percentage reduction from baseline in lesion counts versus minocycline alone at 8 weeks for both inflammatory (−74.4% vs −53.3%; *P* < .001) and noninflammatory lesions (−61.7% vs −42.4%; *P* < .001). The significant reduction in inflammatory lesions was observed as early as week 2 and persisted until the study end. IGA score was significantly lower for minocycline plus PDT versus minocycline alone from as early as week 2 (2.7 vs 3.1; *P* < .01) and persisted until week 8 (1.6 vs 2.3; *P* < .001). In addition, significantly more patients treated with minocycline plus PDT versus minocycline alone achieved an IGA score of “clear” or “almost clear” (success criterion: IGA score <2) at the study end (week 8: 62.5%, 30/48 vs 42.6%, 20/47; *P* < .05) (Fig. 3). Figure 4 illustrates the response after treatments with minocycline plus PDT.

### 3.2 Influence on QOL

**Figure 5:**
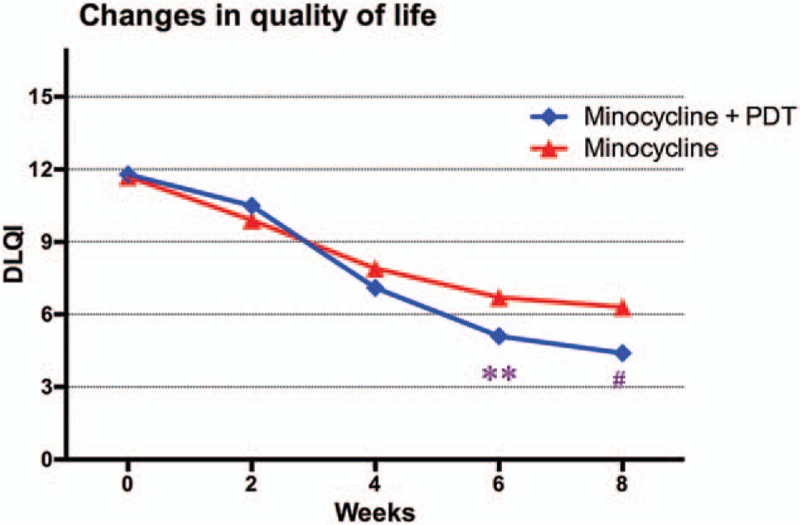
Changes in quality of life according to Dermatology Life Quality Index (DLQI). ^∗^*P* < .01, ^#^*P* < .001.

There were no significant differences in DLQI between the minocycline plus PDT group (11.8 ± 4.7) and the minocycline group (11.7 ± 4.5) at beginning (*P* > .05). As time went on, the DLQI lowered in both groups with a first quick back slow trend, which was in accordance with the changes of acne severity measured by reduction in lesions and the IGA score. At the first follow-up (2 weeks), the DLQI of patients treated with minocycline plus PDT was slightly higher, but the difference had no statistical significance (*P* > .05). After that, the DLQI was significantly lower for minocycline plus PDT compared with minocycline alone and persisted until the last follow-up (week 8: 4.4 vs 6.3; *P* < .001). Changes in patients’ QOL assessed by DLQI are shown in Fig. 5.

### 3.3 Safety evaluation

No severe adverse events were reported in either group. In the minocycline group, 4 patients complained of dizziness, 3 patients presented with a mild headache, and all these symptoms were tolerable (Table 2). In minocycline plus PDT group, pain immediately after PDT was mild and subsided after a few minutes. At the first PDT treatment, median pain was low (2.8, range 0–8), with a similar score after the second (2.7, range 0–9), third (2.8, range 0–8), and fourth treatments (2.9, range 0–9). The correlation between pain after the first and second treatment was 0.78. Mild to moderate erythema after the first illumination was reported in 17 (35%) patients and the erythema subsided within 1 to 3 days in most patients. Most adverse events in this group occurred during or immediately after illumination, and were usually transient. One patient developed hyperpigmentation and faded away 6 weeks later. None of the patients developed ulcers, infection, purpura, scarring, or other substantial adverse effects.

**Table 2:**
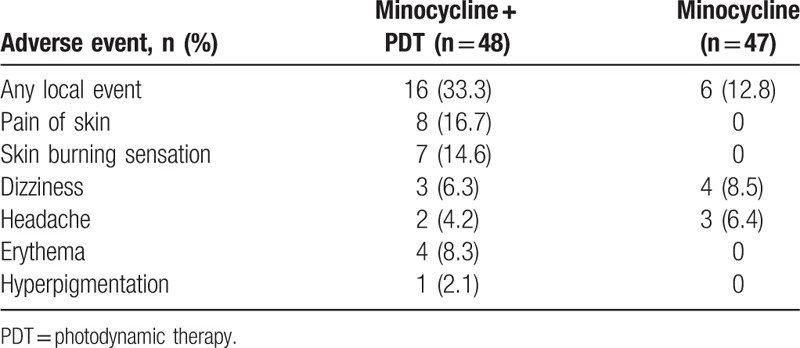
Adverse events reported in follow-ups.

## 4 Discussion

Acne vulgaris, the most common disease in dermatology, brings about not only physical impairments but also psychological burdens, including depression, anxiety, and lower self-esteem.^[18,19]^ Previous treatment for moderate to severe acne mainly relied on systemic use of antibiotics and retinoids; however, these treatments were limited due to adverse effects and less-than-desirable efficacy. Light therapies, especially the PDT, are playing a more and more important role in acne treatment. It offers an alternative to patients who seek topical treatments, a quicker onset of action, nonserious side effects, and decreased antibiotic resistance rates. A variety of PDT protocols, such as ALA + red light, MAL + red light, and ALA + pulsed dye laser, have been used for the treatment of acne.^[20–22]^ The rationale for the use of ALA-PDT in the treatment of acne vulgaris was first investigated by Hongcharu et al^[9]^ by coupling red light and ALA to treat mild to moderate acne. In China, a consensus on ALA-PDT for treatment of acne vulgaris was primarily reached in 2011 by Chinese Dermatologist Association. ALA-PDT has proven to be an effective treatment for acne in several studies.^[23,24]^ However, influence of PDT on patients’ QOL, and the combined effects of PDT and oral medication have rarely been studied.

Antibiotics are still indicated for the treatment of moderate and severe acne. As a second-generation tetracycline, minocycline has good anti-inflammatory property and is not phototoxic compared with doxycycline.^[8]^ It is still a basic medicine in the systemic treatment of moderate to severe acne. Instead of minocycline alone, its combination with PDT might improve the therapeutic effects and provide more treatment options for facial acne patients, especially the severe cases.

We conducted this study to evaluate the efficacy of PDT combined with minocycline against moderate to severe facial acne and influence on QOL by comparing outcomes with those of minocycline alone. In both groups, acne severity graded by IGA score significantly decreased at 8 weeks compared with baseline. Similarly, inflammatory lesions significantly decreased by 74.4% and 53.3% in minocycline plus PDT group and minocycline group, respectively. Interestingly, the mean reduction rate of noninflammatory lesions in both groups (61.7% vs 42.3%) was also significant compared with baseline, though less than that for inflammatory lesions. In addition, significantly more patients treated with minocycline plus PDT versus minocycline alone achieved treatment success defined as IGA score <2 at the last follow-up.

Consistent with lesion reduction and decreased acne severity, patients’ QOL assessed by DLQI improved from 11.8 to 4.4 in minocycline plus PDT group and 11.7 to 6.3 in minocycline group at 8 weeks’ visit. The change was roughly in line with the trend seen in lesion reduction and treatment success rate measured by IGA score. After an initial mild improvement at the first visit after 2 weeks, QOL continued to improve throughout the study period. Patients’ QOL improved as the inflammatory and noninflammatory lesions decreased and the IGA score lowered, which conformed to the results by Gollnick et al.^[17]^ The substantial correlation between acne severity and QOL warranted more specific studies.

In this study, serious adverse effects, such as bulla, crust, and permanent hyperpigmentation, were not observed in either group. It was reported in previous studies that pain was an important issue; however, our study showed that pain was mild to moderate, transient, tolerable, and manageable by short pauses of illumination in most cases. There was a good correlation between the first and second treatments in terms of pain score, indicating that the tolerance of pain in the first treatment was a good sign of tolerance to the subsequent treatments.

However, we recognize several limitations. First, there is no consensus on quantitative evaluation of acne severity at present.^[25]^ Therefore, we used the IGA scale, which was recommended by the US Food and Drug Administration, as an evaluation criterion to judge overall acne severity.^[13]^ Apart from this, a variety of methods have been described to assess QOL in acne patients, with no one widely acknowledged. Additionally, as acne is a long-term disease, longer follow-up is essential to identify the duration of treatment response and permanent effects on facial scarring.

## 5 Conclusions

Amelioration of clinical symptoms brings about corresponding improvement in QOL among facial acne patients. The combination of PDT with minocycline significantly improved clinical efficacy and QOL compared with minocycline alone in patients with moderate to severe facial acne.
